# Anatomic and Cellular Niches for *Mycobacterium tuberculosis* in Latent Tuberculosis Infection

**DOI:** 10.1093/infdis/jiy579

**Published:** 2018-10-30

**Authors:** Jonathan Mayito, Irene Andia, Mulugeta Belay, David A Jolliffe, David P Kateete, Stephen T Reece, Adrian R Martineau

**Affiliations:** 1 Department of Immunology and Molecular Biology, School of Biomedical Sciences, Makerere University College of Health Sciences, Kampala, Uganda; 2 Centre for Immunobiology, Blizard Institute, Barts and The London School of Medicine and Dentistry, Queen Mary University of London, United Kingdom; 3 Kymab Ltd, Babraham Research Campus, Cambridge, United Kingdom

**Keywords:** CD34, bone marrow, hematopoietic stem cells, latent tuberculosis, mesencymal stem cells

## Abstract

Latent tuberculosis has been recognized for over a century, but discovery of new niches, where Mycobacterium tuberculosis resides, continues. We evaluated literature on M.tuberculosis locations during latency, highlighting that mesenchymal and hematopoietic stem cells harbor organisms in sensitized asymptomatic individuals.

## Teaser

Studies suggest that mesenchymal and hematopoietic stem cells may be niches for *M tuberculosis* during latent infection. We propose that propagation of infection by these cells may contribute to anatomical and cellular distribution of *M tuberculosis* in latently infected individuals.

Latent tuberculosis infection (LTBI) is characterized by the presence of immune sensitization to *Mycobacterium tuberculosis* (MTB) in the absence of any clinical or radiological evidence of active disease. The term was coined in 1909 [1], and it has since been validated by molecular studies demonstrating reactivation of disease 33 years postexposure [2]. A recent modeling study estimated that approximately 1.7 billion people have LTBI [3]. Given that LTBI has been recognized for more than a century, and that approximately 1 in 4 people worldwide are affected, it is perhaps surprising that new data relating to the anatomical and cellular niches occupied by MTB during latent infection continue to emerge. For much of the 20^th^ century, MTB was assumed to be confined to macrophages in quiescent granulomas in latently infected individuals. This paradigm has subsequently been challenged by findings from necropsy studies in individuals without signs of active tuberculosis (TB) that report the presence of MTB deoxyribonucleic acid (DNA) in diverse nonphagocytic cell types within and outside the lung [4–6]. Most recently, MTB has been demonstrated in mesenchymal and hematopoietic stem cells [7–10], implicating the bone marrow as a previously unappreciated niche for the organism in LTBI. In this article, we review the historical and contemporary literature relating to anatomical and cellular locations for MTB during latent infection, and we contextualize the presence of MTB in stem cell populations within the bone marrow as well as in differentiated cell types in multiple tissues for maintenance of LTBI in the human host.

## ANATOMIC NICHES

### Necropsy Studies

The earliest evidence relating to anatomic niches for MTB during latent infection comes from historic necropsy studies in which macroscopically normal tissue from individuals who had died of causes other than TB was inoculated into laboratory animals. Findings of these historic studies are summarized in Table 1. Material from lymph nodes (thoracic, cervical, mesenteric and retroperitoneal) and lung (apices and bases) was harvested from autopsies on a total of 628 individuals aged from infancy to old age, and MTB was isolated from animals inoculated with tissues of 96 individuals (15.3%). Two additional studies in which tissue from Ghon foci of individuals without lesions of active TB were inoculated into laboratory animals yielded contrasting results: one study reported the presence of MTB in up to 33% of such lesions [11], whereas another reported a much lower prevalence of just 1.5% [12]. These historic studies have significant methodological limitations: active TB may have been missed in some individuals, resulting in an overestimate of the prevalence of cultivable TB in latent infection. In contrast, inoculated material might have contained small numbers of viable bacilli that did not cause disease in laboratory animals, resulting in an underestimate of prevalence. These limitations notwithstanding, the studies presented in Table 1 were the first to indicate that MTB may reside in macroscopically normal tissue as well as in Ghon foci. This observation paved the way for necropsy studies in the modern era that used polymerase chain reaction (PCR) to demonstrate presence of MTB DNA (which might equally represent the presence of dead or viable bacilli) in diverse cell types in the lung, spleen, liver, kidney, and adipose tissue [4–6]; findings from these studies relating to specific cellular niches for MTB during latent infection are discussed below.

**Table 1.** Results of Historical Studies in Which Macroscopically Normal Tissue From Individuals Dying From Causes Other Than Tuberculosis Was Inoculated Into Laboratory Animals

Reference	Subjects	Material Inoculated	Proportion Culture-Positive
Loomis 1890, cited in [13]	30 adults	Bronchial lymph nodes	8 of 30 (26.7%)
Pizzini 1892, cited in [13]	30 adults	Bronchial and cervical lymph nodes	12 of 30 (40.0%)
Kälble 1899, cited in [13]	23 individuals	Bronchial lymph nodes	2 of 23 (8.7%)
McFadyean 1903, cited in [14]	20 individuals	Mesenteric lymph nodes	2 of 20 (10.0%)
Rosenberger 1905, cited in [14]	14 adults and children	Mesenteric lymph nodes	6 of 14 (42.9%)
Harbitz 1905, cited in [14]	91 children	Cervical, tracheal, mesenteric, and retroperitoneal lymph nodes	18 of 91 (19.8%)
Ipsen 1906, cited in [14]	74 children	Material included mesenteric lymph nodes	1 of 74 (1.4%)
Bartel 1906, cited in [14]	68 children	Cervical, bronchial, and mesenteric lymph nodes	8 of 68 (11.8%)
Weber 1907, cited in [14]	26 children aged 3 months to 12 years	Not ascertained	1 of 26 (3.8%)
Beitzke 1912, cited in [14]	27 children	Cervical, tracheobronchial, and mesenteric lymph nodes	9 of 27 (33.3%)
Eastwood 1914, cited in [14]	61 children	Bronchial, mesenteric, and cervical lymph nodes	5 of 61 (8.2%)
Griffith 1914, cited in [14]	34 children	Bronchial and mesenteric lymph nodes	2 of 34 (5.9%)
Wang [14]	18 adults and 14 children	Cervical, bronchial, mesenteric, and retroperitoneal lymph nodes	3 of 32 (9.4%)
Opie and Aronson [11]	33 adults aged 20–70 years	Tissue from lung apices, lung bases, and hilar or tracheobronchial lymph nodes of individuals with lesions elsewhere (fibrocaseous lesions/scars of apices, caseous encapsulated, or calcified nodes)	15 of 33 (45.5%)
Saenz 1938, cited in [13]	14 individuals	Normal lung	1 of 14 (7.15%)
Feldman and Baggenstoss [13]	51 adults and children aged 2 to 93 years, of whom n = 39 had at least 1 healed Ghon complex	Tissue from upper and lower lobes of the lung and hilar or tracheobronchial lymph nodes	3 of 51 (5.9%)

### Transplant-Transmitted Tuberculosis

Additional evidence supporting presence of MTB in the lung, liver, and kidney of latently infected individuals comes from natural experiments in which tissues from donors with LTBI have been transplanted into immunosuppressed recipients. Pertinent case reports are summarized in Table 2. Although these cannot provide unequivocal proof of disease transmission from latently infected individuals (because subclinical active disease in donors cannot be excluded), they add to the body of evidence suggesting wide anatomical distribution of MTB in latently infected individuals. Case reports of transmission from solid organ transplants are complemented by results of a national survey of TB cases arising in hematopoietic stem cell transplant recipients in Spain [15]: incidence of active TB was significantly higher in recipients of allogeneic versus autologous transplants. This observation is consistent with the hypothesis that hematopoietic stem cells may represent a niche for MTB during LTBI; equally, it might reflect the differential degree of immune compromised status between these 2 groups.

**Table 2.** Case Reports Documenting Potential Transmission of Tuberculosis From Latently Infected Donors to Immunosuppressed Recipients

Reference	Donor	Organ(s) Donated	Recipients
Ridgeway et al [16]	Donor had normal chest radiograph and no known prior history of MTB infection or disease	Lung	Two separate recipients developed pulmonary TB with identical isolate to each other
Graham et al [17]	69-year-old female, died of intracranial hemorrhage, clear chest radiograph, no past history of TB	Kidney and liver (different recipients)	Both recipients developed active TB (renal TB at 14 months posttransplant in kidney recipient, TB osteomyelitis at 12 months posttransplant in liver recipient): matching isolates.
Lee et al [18]	51-year-old, nonsmoking, recent immigrant from China, died of intracerebral hemorrhage. No previous TB, ante-mortem chest x-ray normal, tracheal aspirate smear- and culture-negative for acid-fast bacilli.	Lung	Developed pulmonary MDR TB at 7 weeks; recipient was tuberculin negative pretransplant, with no exposure to MDR-TB.
Boedefeld et al [19]	33-year-old male, died of intracranial haemorrhage, emigrated from Peru 11 years before. Previous PPD test 24 mm, but chemoprophylaxis not given. Chest radiograph normal at time of organ donation.	Lung	Recipient developed pulmonary and pericardial TB at 3 months posttransplant; no known TB exposure.
Kumar et al [20]	42-year-old Vietnamese-born male, died of acute intracranial hemorrhage. No history of TB or positive TST. Ante- mortem CT chest scan showed no pulmonary infiltrates or granulomata.	Lung	TST-negative recipient developed pulmonary TB at 3 months posttransplant; isolate of indo-oceanic lineage, associated with Vietnam/Cambodia.
Mortensen et al [21]	Male in 20s, died in accident; previous incarceration; clear chest radiograph and normal bronchoscopy ante-mortem.	Lung	TST-negative recipient developed pulmonary TB at 2 months posttransplant. MTB isolate matched strain from previous outbreak near donor’s home.
Mortensen et al [21]	Male in 20s, died in accident; previous travel to Philippines; clear chest radiograph, normal bronchoscopy and BAL culture negative for TB ante-mortem.	Lung	Recipient developed PTB at 4 months posttransplant; spoligotype “associated with Manila family,” recipient had not traveled outside of the United States.
Jensen et al [22]	Donor diagnosed with latent TB 5 years before death after exposure to index case with isoniazid-resistant TB; received inappropriate treatment with single agent isoniazid.	Lung	Recipient developed pulmonary TB at 11 weeks posttransplant. MTB isolates from the index case (to whom donor was exposed) and transplant recipient matched.
Cassir et al [23]	47-year-old male, died of intracranial hemorrhage, no risk factors for TB other than chronic alcohol use and smoking. TST results unavailable. No signs of active or previous TB on ante-mortem CT chest. Pretransplantation lung biopsy culture- and PCR-negative for MTB.	Lung	41-year-old female with cystic fibrosis developed pulmonary TB at 6 weeks posttransplant. No previous TB or known TB exposure pre- or posttransplant.
Ruijter et al [24]	57-year-old woman from the Philippines, lethal brain injury. Ante- mortem abdominal ultrasound and chest radiography showed no abnormalities.	Liver	Developed hepatic TB at 6 months posttransplant; MTB isolate Manila family.

Abbreviations: BAL, bronchoalveolar lavage; CT, computed tomography; MDR-TB, multidrug-resistant tuberculosis; MTB, *Mycobacterium tuberculosis*; PCR, polymerase chain reaction; PPD, purified protein derivative; PTB, pulmonary tuberculosis; TB, tuberculosis; TST, tuberculosis skin test.

### Imaging Studies

Further clues relating to the anatomic localization of MTB during latent infection come from studies in which individuals with LTBI have undergone positron emission tomography-computed tomography (PET-CT) scanning—a functional imaging technique that can locate and quantitate uptake of a radiolabeled tracer such as ^18^F-fludeoxyglucose (FDG) by tissues with increased metabolic activity. In one such study, 5 asymptomatic adults with positive interferon-gamma release assay (IGRA) results and normal chest radiographs (human immunodeficiency virus [HIV] status not reported) underwent PET-CT scanning before and 3–4 months after initiation of treatment with isoniazid or rifampin [25]. At baseline, 4 of 5 subjects exhibited increased FDG uptake in hilar, paratracheal, and/or subcarinal lymph nodes—none of which met radiologic criteria for enlargement. The FDG uptake by lymph nodes was decreased at follow-up in 3 of the 4 individuals who exhibited increased uptake at baseline. More recently, Esmail et al [26] identified PET-hot lesions in 10 of 35 HIV-infected adults with a diagnosis of latent TB infection. These lesions were located within pulmonary infiltrates and scars, and 4 of 10 individuals developed active TB during the following 6 months, suggesting that at least some of these lesions may have represented early active TB rather than stable latent infection.

Taken together, these studies indicate that MTB has a wide anatomical distribution in latently infected individuals, and that it may be found in both macroscopically normal and lesional tissue, both within and outside the lung. Additional support for the concept that MTB may reside at extrapulmonary sites comes from studies reporting reactivation of TB at wide anatomical locations after biological immunotherapy and HIV infection [27–29]. Before reviewing the literature relating to specific intracellular bacillary niches, we next consider the possibility of an extracellular location for MTB during latent infection.

## AN EXTRACELLULAR NICHE?

Persistence of extracellular MTB during LTBI is unproven, but there is a widely held belief that at least some bacilli may survive in extracellular caseous material within granulomas during latency, unable to multiply because of the hypoxic and proteolytic microenvironment [30]. A study in mice showed that encapsulation of MTB in hollow fibers inserted subcutaneously resulted in infiltrating cells forming granulomatous lesions around the inserted fibers. Mycobacteria contained within the fibers tuned down patterns of gene expression commensurate with active growth and upregulated expression of dormancy genes, suggesting that induction of dormancy can occur in extracellular organisms [31]. Furthermore, mycobacteria are able to use extracellular hyaluronan as a carbon source for growth, suggesting that they are able to persist outside host cells, at least temporarily [32]. Whether this phenomenon plays a role in latency remains to be established. More recent studies have demonstrated that MTB releases mycobacterial extracellular vesicles that exert diverse effects on the host response, including induction of Toll-like receptor (TLR)-2 signaling [33]. Although this may augment anti-mycobacterial responses in the short term, sustained TLR-2 stimulation may drive anti-inflammatory responses and inhibit Th1 polarization of CD4^+^ T cells [34]; such regulatory actions could favor extracellular mycobacterial persistence. MTB-infected neutrophils also release extracellular vesicles that activate macrophages and promote the clearance of intracellular MTB through early superoxide anion production and autophagy induction [35].

## NICHES IN PROFESSIONAL PHAGOCYTES

### Macrophages

The macrophage is the primary intracellular habitat for MTB during active disease: it therefore represents an obvious candidate as a niche during latency. Understanding the potential role of the infected macrophage in latency is complicated by the fact that 2 distinct macrophage lineages have been described in the lung, both of which are infected by MTB: alveolar macrophages are derived during embryogenesis from fetal liver and are capable of self-renewal at steady state, whereas interstitial macrophages are generally thought to arise from blood-derived monocytes at steady state. These ontologically distinct populations have been reported to differ in their responses to MTB: alveolar macrophages upregulate fatty acid uptake and β-oxidation to provide a more nutritionally permissive environment for MTB, whereas interstitial macrophages are highly glycolytically active to exert nutritional restriction and control bacterial growth [36]. *Mycobacterium tuberculosis* itself can influence development of macrophage phenotype: Peyron et al [37] used an in vitro granuloma model to show that MTB long-chain fatty acids triggered differentiation of monocyte-derived macrophages into longer-lived foamy macrophages, characterized by the presence of lipid-containing bodies, that exhibited diminished intracellular bactericidal activity and harbored intracellular MTB in a dormant, nonreplicative state. These organisms adapted their metabolic strategy to use accumulated cytoplasmic lipid bodies, in particular triacylglycerol, as both carbon and energy sources. This metabolic shift was associated with upregulated mycobacterial expression of the gene encoding isocitrate lyase, an enzyme that is essential for metabolism of fatty acids and persistence of MTB in macrophages [38]. Foamy macrophages could also provide a long-term niche for dormant MTB by affording bacilli access to host cholesterol, which is also required for mycobacterial persistence [39]. In addition, Singh et al [40] have reported that MTB induces the foamy macrophage phenotype, via targeted manipulation of host cellular metabolism to divert the glycolytic pathway toward ketone body synthesis, which led to perturbations in lipid homeostasis and consequent accumulation of lipid bodies in the macrophage. Studies of *Mycobacterium marinum* infection in a zebra fish model showed that intracellular mycobacteria can induce recruitment of new macrophages to nascent primary granulomas, which then “seed” secondary granulomas via egress of infected macrophages [41]. Therefore, macrophages may play a key role in systemic dissemination of mycobacteria, as well as provide potential niches for bacilli in the lung during latent infection.

### Dendritic Cells

Using murine infection, Wolf et al [42] showed (1) that up to 80% of MTB bacilli in the lung and draining lymph nodes of infected animals were found in dendritic cells and (2) that presence of MTB in dendritic cells and subsequent presentation of antigen to CD4^+^ T cells was critical for the development of protective immune responses [43]. The latency-associated MTB proteins Hip1 (hydrolase important for pathogenesis 1, a cell envelope-associated serine hydrolase) and Acr1 (α-crystallin 1, a small heat shock protein) have both been shown to impair dendritic cell function [44, 45]: tuning down of dendritic cell function by latent MTB could therefore facilitate bacterial persistence during LTBI. Monocyte-derived inflammatory dendritic cells are the dominant dendritic cell type in the chronic granuloma [46]: they are more migratory compared with macrophages, and they are more efficient in transporting antigens to lymph node T cells [47]. *Mycobacterium tuberculosis* might therefore exploit the migratory capacity of dendritic cells to promote dissemination during latency. However, the role of dendritic cell carriage of MTB in long-term persistence remains unclear.

### Neutrophils

Neutrophils are among the first phagocytic cells at the site of MTB infection and are present in granulomatous lesions during both acute and chronic TB in mouse models [48, 49]. They have been shown to transport live mycobacteria from peripheral tissue to lymphoid organs in mice, surviving longer and having increased capacity for migration when harboring intracellular mycobacteria [50]. *Mycobacterium tuberculosis* inhibits neutrophil apoptosis, and abrogation of this effect by deletion of the MTB NuoG gene (which encodes a subunit mycobacterial type I NADH dehydrogenase complex) increases apoptosis of MTB-infected neutrophils with subsequent cross-priming to dendritic cells and accelerated CD4^+^ T-cell priming [51]. Because neutrophils are ultimately short-lived—even with increases in lifespan induced by mycobacterial infection [52]—it appears unlikely that they represent a niche for MTB carriage in LTBI. More probably, they are harnessed by mycobacteria for early dissemination.

## OTHER INTRACELLULAR NICHES

The principal line of evidence implicating nonmyeloid cells as potential niches for MTB during latent infection comes from 3 studies mentioned above that used PCR to detect MTB DNA in necropsy samples from individuals who had died from causes other than TB. Hernández-Pando et al [4] detected MTB DNA in sections of macroscopically normal lung tissue from 5 of 13 such individuals from Ethiopia and 10 of 34 from Mexico. Positive cells included alveolar and interstitial macrophages, type II pneumocytes, endothelial cells, and fibroblasts. Neyrolles et al [5] detected MTB DNA in perinephric, perigastric, pericardial, and subcutaneous adipose tissue in 6 of 19 individuals from Mexico and 6 of 20 individuals from France. Barrios-Payán et al [6] studied 49 individuals in Mexico, and detected MTB DNA in the lung (36 of 49 individuals), the spleen (35 of 49), the kidney (34 of 49), and the liver (33 of 49). Affected cells included endothelium, pneumocytes, and macrophages from the lung, Bowman’s parietal cells, and convoluted proximal tubules from the kidney, macrophages, and sinusoidal endothelial cells from the spleen, and Kupffer cells and sinusoidal endothelium in the liver. It should be noted that MTB in these cells was noncultivable and therefore unlikely to fulfill Koch’s postulates. Although nonreplicating MTB has been shown to express 16S ribosomal ribonucleic acid and latency-associated genes encoding isocitrate lyase and α-crystallin [53, 54], evidence is lacking to show that nonreplicating organisms can be resuscitated to cause active disease. The uncoupling of the presence of MTB versus its ability to cause active disease represents a significant challenge for contextualization of these findings for LTBI. However, data from tissue culture studies and animal models provide additional evidence that specific nonmyeloid cells may act as niches for MTB during latent infection. These nonprofessional phagocytic cells are particularly well suited as niches for MTB persistence because they can provide a conducive and protective intracellular environment that enables them to evade recognition by the host immune system.

### Respiratory Epithelial Cells

*Mycobacterium tuberculosis* has been shown to invade type II alveolar epithelial cells by utilizing heparin-binding hemagglutinin, TLRs, and surfactant proteins [55], and to exhibit a distinct transcriptional profile while replicating within them, with upregulation of genes encoding virulence factors and downregulation of hypoxia-induced genes [56]. Using a murine chronic infection model, Rivas-Santiago et al [57] showed (1) that ciliated and nonciliated bronchial epithelial cells could be infected by MTB and (2) that these cells produced higher levels of the antimicrobial peptide β-defensin than macrophages in response to infection. These findings are consistent with results from experiments conducted by the same group in a human lung epithelial cell line, in which control of MTB growth by alveolar epithelial cells was associated with induction of β-defensin expression by lipoarabinomannan, a component of the MTB cell wall [58]. The authors of these studies hypothesized that β-defensins could exert bacteriostatic effects on MTB within respiratory epithelial cells to limit bacillary proliferation and induce dormancy. Using a 2-layer Transwell system, Bermudez et al [59] found that replication of MTB within a type II alveolar cell line enhanced its ability to subsequently invade endothelial cells. Moreover, they showed (1) that MTB-infected monocytes could translocate across a bicellular layer of epithelial and endothelial cells and (2) that this process was more efficient when the epithelial cells were themselves infected with MTB [59]. Taken together, these studies indicate that alveolar epithelial cells represent a conducive environment for MTB persistence as well as a niche from which MTB can expand and disseminate from the lung before elicitation of adaptive immune responses.

### Fibroblasts

*Mycobacterium tuberculosis* can infect and replicate in fibroblasts, which are able to present antigen from heat-killed MTB via major histocompatibility complex-II [60]. Fibroblasts potentiate bactericidal effects in both nonactivated and activated macrophages through increased nitrite production; these effects are more pronounced when fibroblasts themselves are coinfected with MTB [61]. These findings point to an active role of fibroblasts in protection within granulomas; however, carriage of MTB within fibroblasts during LTBI remains in question.

### Lymphatic Endothelial Cells

*Mycobacterium tuberculosis* disseminates from the lungs to extrapulmonary sites via lymphatic and circulatory systems. Lymph nodes are the most common sites for extrapulmonary TB, with viable bacilli often recovered from these sites [62]. *Mycobacterium tuberculosis* has been shown to infect lymphatic endothelial cells and to egress to the cell cytosol using MTB cell wall phthiocerol dimycocerosates to lyse autophagosomes [63]. When activated by interferon-γ, lymphatic endothelial cells use autophagy and nitric oxide to kill intracellular bacteria. However, when inactivated, they can support MTB growth and persistence [64].

### Adipocytes

Neyrolles et al [5] showed that MTB infects adipocytes via scavenger receptors, with intracellular bacilli observed within membrane-bound vacuoles. Intracellular MTB accumulated in cytoplasmic lipid droplets, with this effect becoming more pronounced in mature adipocytes. Furthermore, intracellular MTB was shown to replicate in fibroblast-like preadipocytes, with replication decreasing as preadipocytes differentiated into mature adipocytes [5]. Other investigators have reported that infection of adipose tissue by MTB is accompanied by infiltration of natural killer cells and MTB-specific CD8^+^ T cells and upregulation of tumor necrosis factor, interleukin (IL)-6, adiponectin, and IL-10 [65, 66]. In contrast to many of the cell phenotypes thus far considered as intracellular niches for LTBI, adipocytes are relatively long-lived in the host, with a life span of 8 years or more [67].

### Stem Cells

Recent studies have shown that both mesenchymal and hematopoietic stem cells recovered from individuals with LTBI or having successfully received chemotherapeutic treatment for active TB contained intracellular MTB in a predominantly uncultivable form. The hypoxic environment of the bone marrow [68, 69], expression of active drug efflux pumps by bone marrow stem cells, and the lack of intracellular antibacterial mechanisms in early-stage stem cells [70, 71] all conspire to make bone marrow stem cells an attractive potential niche for carriage of MTB during latent infection. Das et al [7] showed (1) that mesenchymal stem cells from healthy volunteers were preferentially infected by virulent MTB in vitro and (2) that viability of MTB dropped 4-fold when the mesenchymal stem cells lost their CD271/CD133 markers, indicating that the undifferentiated mesenchymal stem cell state favors persistence of infection. *Mycobacterium tuberculosis* infected the CD271^+^ bone marrow mesenchymal stem cells via scavenger receptors MARCO and SR-B1, and mesenchymal stem cells controlled intracellular MTB using autophagic mechanisms [72]. Mesenchymal stem cells killed avirulent mycobacteria but not MTB via modulation of cathelicidin expression [73]. Moreover, CD271^+^ bone marrow mesenchymal stem cells harboring MTB are localized in the hypoxic niche in both mice and humans, a critical microenvironmental factor that is known to induce dormancy [74]. The ability of mesenchymal stem cells to maintain MTB depends on the inflammatory milieu: Yang et al [75] reported that murine macrophages produced cytokines during mycobacterial infection that promoted clearance of MTB from mesenchymal stem cells by increasing production of nitric oxide in an IL-1β-dependent manner.

Tornack et al [10] reported that human peripheral blood long-term pluripotent hematopoietic stem cells harvested from IGRA-positive asymptomatic individuals contained MTB DNA, whereas those from IGRA-negative individuals did not. *Mycobacterium tuberculosis* within long-term pluripotent hematopoietic stem cells expressed dormancy genes and did not form colonies on agar but were resuscitated when administered intratracheally to immune-deficient *Rag*^*-/*-^*IL2Rγ*^*-/*-^ mice to form nascent lung granulomas accompanied by detection of cultivable MTB. Using a murine infection model, Reece et al [9] showed that hematopoietic stem and progenitor cells containing noncultivable MTB propagated canonical hallmarks of TB when transferred to recipient naive mice, provided that both donor and recipient mice were unable to express inducible nitric oxide synthase 2. Nitric oxide synthase 2 mediates killing of intracellular bacteria via production of nitric oxide, a key protective mechanism against TB in mice. Nitric oxide synthase 2 is not expressed in nondifferentiated hematopoietic stem cells, and hence MTB could remain viable in them [76].

**Figure 1.**
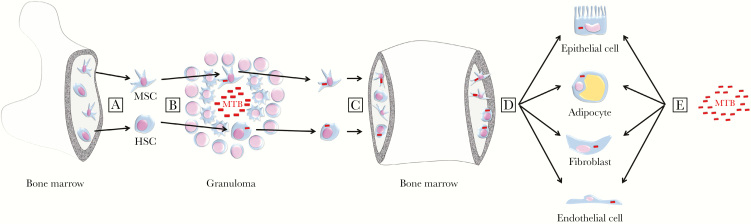
Hypothesis: bone marrow stem cells seed *Mycobacterium tuberculosis* infection to the periphery. (A) Uninfected mesenchymal stem cells (MSC) and hematopoietic stem cells (HSC) enter the circulation from the bone marrow. (B) The cells migrate to, and are incorporated into, the granuloma where they are infected with *M tuberculosis* (red bars). (C) Infected MSC and HSC return to the bone marrow to establish a niche of *M tuberculosis* infection during latency. (D) Infected stem cells propagate *M tuberculosis* infection to the periphery. (E) *Mycobacterium tuberculosis* may also infect nonmyeloid cells directly, eg, via the airway or by hematogenous or lymphatic spread.

Both mesenchymal and hematopoietic stem cells are highly mobile, and they transition between the bone marrow, the circulation, and TB granulomas [77]. In Figure 1, we illustrate the hypothesis that stem cells containing dormant MTB could therefore seed new infectious foci in peripheral tissues. It is intriguing that almost all the cell types discussed in our review as potentially harboring MTB can differentiate from progenitor states of either mesenchymal or hematopoietic stem cells (the exception being tissue-resident macrophage lineages, which are seeded during waves of embryonic hematopoiesis and self-maintained independent of contribution from the bone marrow during adulthood [78]). This observation raises the possibility that MTB in differentiated cells within the tissues could originate from a precursor stem cell that already contained MTB. The fact that hematopoietic stem and progenitor cells harboring MTB from both experimentally infected mice and humans with LTBI can propagate TB after adoptive transfer of cells in mouse models represents an important starting point in testing this hypothesis. Further investigation is needed to track the developmental states of the transferred cells harboring MTB as infection develops. In considering this hypothesis, we raise 2 important caveats. First, we do not exclude the probability that some nonmyeloid cell types (particularly epithelial and endothelial cells) may become infected directly as well as or instead of via a stem cell route. Second, we acknowledge that these niches are unlikely to be specific to the context of latent infection, and that infection of all of these different cell types may also occur during active TB, which typically arises within 2 years of exposure to an infectious case [79].

## CONCLUSIONS

The presence of MTB in diverse nonphagocytic cells of latently infected individuals has been recognized for almost 20 years, but the route by which the bacillus reaches these sites has not been apparent. Recent discoveries implicating hematopoietic and mesenchymal stem cells as potential niches for MTB raise the possibility that stem cells may disseminate infection, leading to the wide anatomical and cellular distribution of MTB reported in latently infected individuals. Understanding the role of both hematopoietic and mesenchymal stem cells in the maintenance of latent MTB infection and triggering of active TB disease remains an exciting area of investigation.

## Notes

***Disclaimer*.** The views expressed in this publication are those of the author(s) and not necessarily those of the Medical Research Council, African Academy of Sciences (AAS), New Partnership for Africa’s Development Planning and Coordinating Agency (NEPAD), Wellcome Trust, or the United Kingdom government.

***Financial support.*** This work was supported by grants from the Medical Research Council (ref. MR/P024548/1; to A. R. M.) and the DELTAS Africa Initiative (ref. DEL-15-011 [to J. M.], via THRiVE-2). The DELTAS Africa Initiative is an independent funding scheme of the AAS’s Alliance for Accelerating Excellence in Science in Africa and supported by the NEPAD Agency with funding from the Wellcome Trust Grant No. 107742/Z/15/Z and the United Kingdom government.

***Potential conflicts of interest.*** S. T. R. has a pending patent that is relevant to the work. All authors have submitted the ICMJE Form for Disclosure of Potential Conflicts of Interest. Conflicts that the editors consider relevant to the content of the manuscript have been disclosed.
